# Cyclin B1 Destruction Box-Mediated Protein Instability: The Enhanced Sensitivity of Fluorescent-Protein-Based Reporter Gene System

**DOI:** 10.1155/2013/732307

**Published:** 2013-12-15

**Authors:** Chao-Hsun Yang, Wan-Ting Kuo, Yun-Ting Chuang, Cheng-Yu Chen, Chih-Chien Lin

**Affiliations:** ^1^Department of Cosmetic Science, Providence University, No. 200, Section 7, Taiwan Boulevard, Shalu District, Taichung 43301, Taiwan; ^2^Graduate Institute of Biotechnology, National Chung Hsing University, 250 Kuo-Kuang Road, Taichung 40227, Taiwan

## Abstract

The periodic expression and destruction of several cyclins are the most important steps for the exact regulation of cell cycle. Cyclins are degraded by the ubiquitin-proteasome system during cell cycle. Besides, a short sequence near the N-terminal of cyclin B called the destruction box (D-box; CDB) is also required. Fluorescent-protein-based reporter gene system is insensitive to analysis because of the overly stable fluorescent proteins. Therefore, in this study, we use human CDB fused with both enhanced green fluorescent protein (EGFP) at C-terminus and red fluorescent protein (RFP, DsRed) at N-terminus in the transfected human melanoma cells to examine the effects of CDB on different fluorescent proteins. Our results indicated that CDB-fused fluorescent protein can be used to examine the slight gene regulations in the reporter gene system and have the potential to be the system for screening of functional compounds in the future.

## 1. Introduction

**Figure 1:**
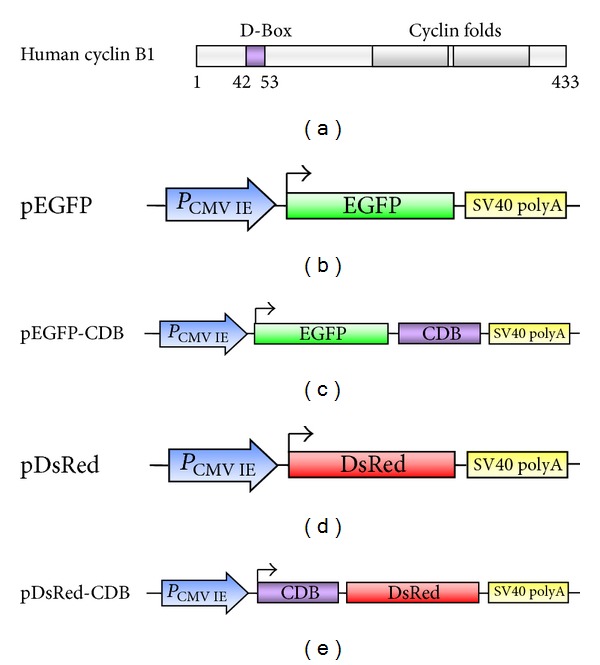
Illustrations of (a) human cyclin B1 destruction box (CDB; D-Box), (b) pEGFP plasmid, (c) pEGFP-CDB plasmid, (d) pDsRed plasmid, and (e) pDsRed-CDB plasmid. The reporter genes were controlled by CMV IE promoter.

The correctly regulated cell cycle requires the periodic expression and destruction of several cyclins [1, 2]. In mammalian cells, special cyclin-cyclin-dependent kinase (Cdk) complexes control different cell cycle transitions. For example, cyclin D-Cdk4/6 regulates G1 phase progression, cyclin E-Cdk2 controls the G1-S transition, and cyclin B-Cdk1 makes confirmation of M phase entrance [3, 4]. Level of cyclin B1 is firstly augmented during the G2 phase, which allows the accumulation of cyclin B1-Cdk1 complexes. Cyclin B1-Cdk1 activation contributes to the separation of centrosomes in late G2 phase [5]. After requirements for the cell cycle checkpoint have been achieved, the progressive loss of cyclin B1-Cdk1 activity, cyclin B1 degradation, and Cdk1 inactivation is necessary for successful chromosome segregation and completion of cell division [6, 7]. Cyclin is degraded by the ubiquitin-proteasome system. The ubiquitin-proteasome system consists of a nonspecific ubiquitin-activating enzyme (E1), an ubiquitin-carrier protein (E2), a specific ubiquitin ligase (E3), and a constitutively active proteasome complex [4, 8]. The major cell cycle ubiquitin ligase for cyclin B is the anaphase-promoting complex/cyclosome (APC/C) complex [9]. In addition, a short sequence near the N-terminal of cyclin B called the destruction box (D-box; CDB) is also required. Full-length human cyclin B1 contains 433 amino acids and the D-box (CDB) with a feature of RXXL sequence can be found at the amino acid sequence from 42 to 53 (Figure 1(a)) [8, 10]. The predicted full amino acid sequence of human CDB is RTALGDIGNKVS.

Regulations of genes are often difficulty observed in cells. Therefore, to overcome such problem, the reporter gene system was developed to examine such precise gene regulations in cells [11]. Fluorescent proteins are frequently used in reporter gene system because of their convenience feature with simplicity, rapidity, and clarity [12]. However, fluorescent proteins, such as green fluorescent protein (GFP) and red fluorescent protein (RFP, also named DsRed), are overly stable that causes their insensitivity to analysis [13, 14]. Besides, luciferase and secreted alkaline phosphatase (SEAP) are frequently used enzymes in many applications including reporter gene system [15–17]. Although luciferase is a sensitive enzyme to gene regulation analysis, the analytic procedures are complex and time consuming [18]. Therefore, destabilizing such overstable fluorescent proteins is a good strategy for improvement of reporter gene system [19, 20].

Earlier study has been observed that a mouse cyclin D-box containing protein fragment can reduce the half-life of its fusion protein in mouse cells with the regulation of cyclins in cell cycles [21]. However, the stability variation of human CDB fusion protein was not tested and the regulative effects of human CDB on fluorescent-protein-based reporter gene system in human cells were not evaluated as well. Therefore, in this study, we use human CDB fused with both enhanced green fluorescent protein at C-terminus and red fluorescent protein at N-terminus in the transfected human melanoma cells to examine effects of CDB on different fluorescent proteins. Besides, the correlation between protein expression and its mRNA levels was also confirmed. Moreover, we utilize curcumin as an inducer of cytomegalovirus-immediate early (CMV IE) promoter to investigate the differences of induction ratio between original and CDB-fused fluorescent proteins.

## 2. Materials and Methods

### 2.1. Materials

Fetal bovine serum (FBS), L-glutamine, penicillin-streptomycin, deoxynucleotide triphosphate (dNTP), oligo (dT), *Pfu* and *Taq* DNA polymerase, M-MLV reverse transcriptase, and Modified Eagle Medium (MEM) were purchased from Gibco BRL/Invitrogen (Carlsbad, CA, USA). Curcumin (>95%), ethidium bromide, dithiothreitol (DTT), agarose, and other chemicals were purchased from Sigma-Aldrich (St. Louis, MO, USA). PolyJet Transfection Reagent was purchased from SignaGen Laboratories (Ijamsville, MD, USA). Restriction enzymes were purchased from New England BioLabs (Beverly, MA, USA). Deionized distilled water (ddH_2_O) used to prepare solutions was purified using a Milli-Q system (Millipore, Bedford, MA, USA).

### 2.2. Plasmid Construction

The genomic DNA from human melanoma cells were extracted by FavorPrep Blood/Cultured Cell Genomic DNA Extraction Mini Kit (Favorgen, PingTung, Taiwan). Human cyclin B1 destruction box (CDB, 42 to 53 aa, Figure 1(a)) DNA fragment was amplified through the extracted genomic DNA by *Pfu* DNA polymerase with designed primers (Table 1). The prepared CDB DNA fragment was then cloned into pEGFP-C3 and pDsRed-Express-N1 (Figures 1(b) and 1(d)) vectors (Clontech, Mountain View, CA, USA) by *Sac*II and *BamH*I restriction sites to generate pEGFP-CDB and pDsRed-CDB plasmids (Figures 1(c) and 1(e)).

### 2.3. Cell Culture and Transfection

The human melanoma cell line, MeWo cells (BCRC 60540), was obtained from the Bioresource Collection and Research Center (BCRC, Hsinchu, Taiwan). MeWo cells were cultured in MEM supplemented with 10% FBS, 2 mM L-glutamine, and 1% penicillin-streptomycin (100 U/mL penicillin and 100 *μ*g/mL streptomycin). The cells were maintained in a humidified incubator at 37°C with 5% CO_2_. The cells were subcultured every 3-4 days to maintain logarithmic growth and were allowed to grow for 24 hours before transfection or treatment was applied. For transfection, MeWo cells were cultured in 24-well plates with 1 × 10^5^ cells/well and the prepared plasmids were transfected by PolyJet Transfection Reagent into cells at concentration of 1 *μ*g/well according to the protocol. Transfected cells were analyzed by fluorescent microscopy at each time point to confirm the protein expression and then further measured by ELISA reader or RT-PCR experiment. The transfection efficiency of tested plasmid in the same group (EGFP or DsRed) was measured by the cotransfected control plasmid at the ratio of 10 : 1. For example, in EGFP group, pDsRed plasmid is used as cotransfected control plasmid.

### 2.4. Fluorescent Protein Intensity Measurement

The culture medium of the transfected cells (1 × 10^5^ cells/well) in 24-wells plate were replaced by phosphate buffered saline (PBS) and then the fluorescent intensities were measured using an enzyme-linked immunosorbent assay (ELISA) microplate reader (*μ*Quant, Bio-Tek, GA, USA) at each time point (0 to 96 h). The measurements were set to excitation/emission of 488/507 nm for EGFP and 557/579 nm for DsRed, respectively. The measured intensity was normalized through the intensity of its cotransfected control plasmid and then expressed as relative fluorescent intensity.

### 2.5. Reverse Transcription Polymerase Chain Reaction (RT-PCR)

Primers used for RT-PCR analyses are listed in Table 1. Glyceraldehyde 3-phosphate dehydrogenase gene (GAPDH) is used as an internal control. The reverse transcription (RT) reaction was performed using 5 *μ*g of total RNA, 1 *μ*L of oligo (dT), 1 *μ*L of dNTP mix (10 mM), and up to 12 *μ*L of ddH_2_O. The mixture was heated for 5 min at 65°C and quickly chilled on ice. Subsequently, 4 *μ*L of first strand buffer, 2 *μ*L of 0.1 M DTT, and 1 *μ*L of ribonuclease inhibitor (40 U/*μ*L) were added to the mixture. The mixture was incubated at 37°C for 2 min, and 1 *μ*L of M-MLV reverse transcriptase was added. The reaction was stopped by heating the solution to 70°C for 15 min. A 1 *μ*L aliquot of cDNA mixture was used in the subsequent enzymatic amplification. Polymerase chain reaction (PCR) was performed using 1.5 mM MgCl_2_, 0.2 mM dNTP, 2.5 units of Taq DNA polymerase, and 0.1 *μ*M each of the primers (Table 1). The amplified products were separated in 2% agarose gel in Tris-borate-EDTA (TBE) buffer and stained with ethidium bromide.

### 2.6. Promoter Induction

Curcumin was used as an inducer for cytomegalovirus-immediate early (CMV-IE) promoter controlled reporter genes. The transfected MeWo cells (1 × 10^5^ cells/well) in 24-well plates were treated with or without 5 *μ*M curcumin and then the fluorescent intensities were measured at each time point (0 to 72 h). The induction ratios of all conditions were calculated to analyze the inductivity.

### 2.7. Statistical Analysis

The quantitative data for the present study were analyzed using Student's *t*-tests and are presented as mean ± SE for three independent experiments.

## 3. Results and Discussion

### 3.1. Plasmid Construction and Transfection

Full-length human cyclin B1 contains 433 amino acids and the predicted full amino acid sequence of human CDB is RTALGDIGNKVS (42 to 53 aa, Figure 1(a)). Human CDB DNA fragment was amplified through the extracted human genomic DNA by PCR with designed primers (Table 1). The prepared CDB DNA fragment was then cloned into pEGFP-C3 and pDsRed-Express-N1 (Figures 1(b) and 1(d)) vectors at 3′ end and 5′ end of its reporter gene to generate pEGFP-CDB and pDsRed-CDB plasmids (Figures 1(c) and 1(e)). Therefore, the constructed plasmids can express EGFP-CDB fusion protein and CDB-DsRed fusion protein by the control of CMV IE promoter in transfected human cells.

In this study, we used human melanoma cell line, MeWo cells, as the tested cells to analyze the effect of human CDB on the fluorescent proteins expression. The transfection efficiency in the same group (EGFP or DsRed) was calculated and normalized by the cotransfected control plasmid at the ratio of 10 : 1. Cellular morphology and cell viability of these transfected cells have no difference in our experiments. Besides, the transfection efficiency in each group is also similar (data not shown). The transfected cells were subsequently examined by fluorescent microscopy and ELISA reader to recognize the expression level of these fluorescent proteins.

### 3.2. Fluorescent Intensities of Original and CDB-Fused Fluorescent Proteins

**Figure 2:**
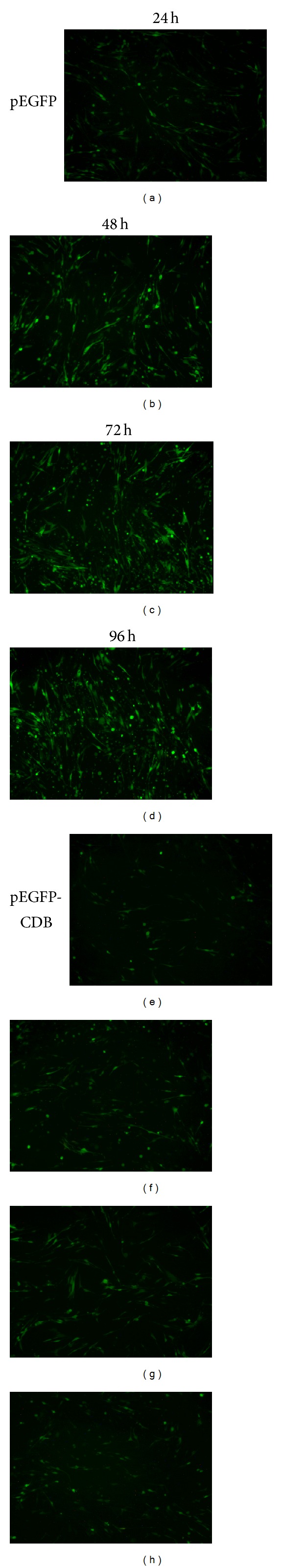
Fluorescent images of pEGFP plasmid ((a) to (d)) and pEGFP-CDB ((e) to (h)) plasmid transfected MeWo cells at different time points.

**Figure 3:**
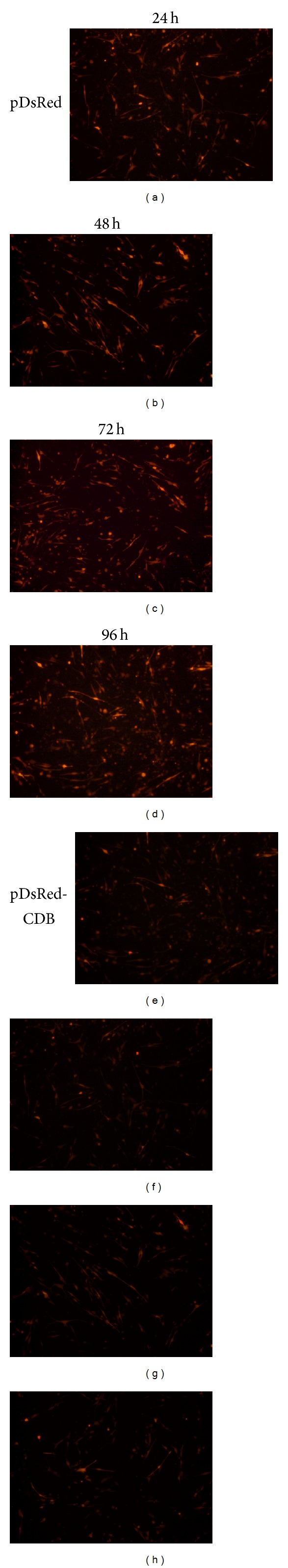
Fluorescent images of pDsRed plasmid ((a) to (d)) and pDsRed-CDB ((e) to (h)) plasmid transfected MeWo cells at different time points.

The fluorescent images of pEGFP/pEGFP-CDB and pDsRed/pDsRed-CDB plasmid transfected MeWo cells at different time points were shown in Figures 2 and 3, respectively. Our results clearly showed that EGFP and EGFP-CDB expression in cells at 24 h have a comparable fluorescent signal. However, by increase of time, from 48 to 96 h, the fluorescent signals of pEGFP transfected cells was evidently higher than those of pEGFP-CDB transfected cells (Figure 2). Moreover, from 24 to 96 h, the fluorescent signals of pEGFP-CDB transfected cells were maintained at an equivalent level. A similar result was shown in Figure 3 for pDsRed and pDsRed-CDB transfected cells; the fluorescent signals of pDsRed transfected cells were also obviously higher than those of pDsRed-CDB transfected cells.

**Figure 4:**
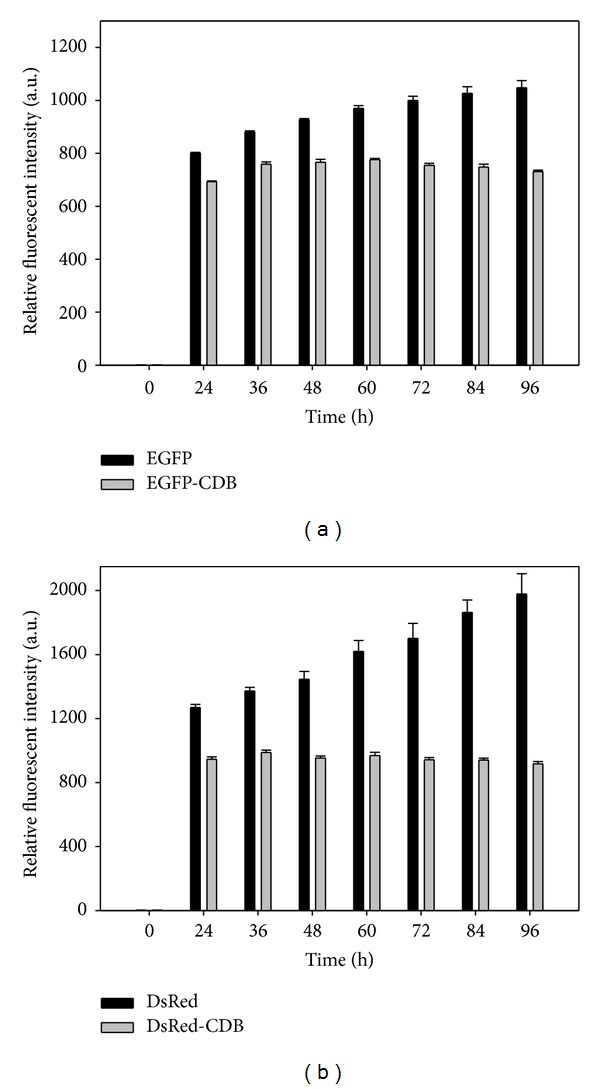
Relative fluorescent intensities of (a) pEGFP/pEGFP-CDB plasmids and (b) pDsRed/pDsRed-CDB plasmids transfected MeWo cells at different time points. Each value is expressed as mean ± SE (*n* = 3).

Fluorescent intensities of these transfected cells at different time points were analyzed through ELISA reader to quantify these fluorescent signals. Results were shown in Figure 4. This result was correlated with that we observed in Figures 2 and 3. During the expression time period, it is noticeable that both EGFP and DsRed (original fluorescent proteins) have great fluorescent intensities in cells. Compared with EGFP-CDB, the fluorescent intensity of EGFP is approximately 45% higher than that of EGFP-CDB at 96 h. In addition, the fluorescent intensity of DsRed is accumulated in cells. When compared with CDB-DsRed at 96 h, the intensity of DsRed is even higher than twofold that of CDB-DsRed (Figure 4). These results demonstrated that the original fluorescent proteins are relatively stable and accumulated in the transfected cells. On the other hand, although CDB-fused fluorescent proteins are also expressed in cells during the period, they cannot constantly stay in cells because the CDB fusion proteins may be destroyed with cyclins by ubiquitin-proteasome system in cell cycle [8]. In addition, D-box is placed near the N-terminus of cyclins (Figure 1(a)); however, the CDB fragment can affect its fusion protein whether it is fused at N-terminal or C-terminal end (Figures 2, 3, and 4). Our study is the first to investigate the effects of CDB on the fused protein at different terminuses.

Although chemiluminescence is more sensitive than fluorescence in nearly all systems, fluorescent reporters still have some advantages; for example, fluorescent proteins were suited to bioimaging assays in living cells which does not require cell permeabilization or the addition of exogenous substrates [18]. Besides, the fluorescent intensity of fluorescent proteins can be associated with its protein expression level [22–24]. Therefore, if the fluorescent proteins are overly stable, there is a limitation for such reporter system to analyze some precise regulations of target gene. To confirm the relationship between fluorescent protein level and its gene regulation, we use RT-PCR assay in the subsequence experiment.

### 3.3. RT-PCR Analyses of Original and CDB-Fused Fluorescent Proteins

**Figure 5:**
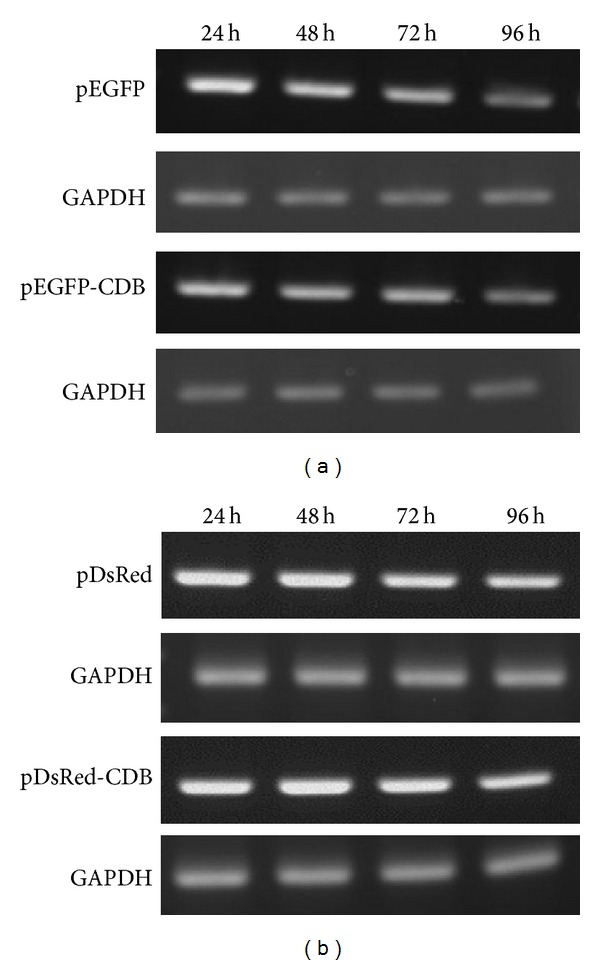
RT-PCR analyses of (a) pEGFP/pEGFP-CDB and (b) pDsRed/pDsRed-CDB plasmids transfected MeWo cells at different time points. Levels of GAPDH mRNA were used as internal control.

The RT-PCR results were shown in Figure 5. For pEGFP transfected cells, the reporter gene mRNA (EGFP mRNA) was obviously amplified by PCR at all time points. However, from 24 to 96 h, the mRNA amounts of reporter gene were decreased with time (Figure 5(a)). This result was quite different from the fluorescent intensities of EGFP in Figures 2 and 4(a). Therefore, these results indicated that original EGFP was expressed via the control of CMV IE promoter and then stably accumulated in cells. Although mRNA levels of EGFP are reduced in cells with time, the overly stable EGFP still makes the fluorescent intensity increase. In contrast, the variation of EGFP mRNA in pEGFP-CDB transfected cells was similar to the results of fluorescent intensities in Figures 3 and 4(b). Because the EGFP-CDB protein is decomposed with cell cycle in mitotic cells, the fluorescent intensity in pEGFP-CDB transfected cells almost reflects its gene regulations. Moreover, similar results were also observed in DsRed system in Figure 5(b). Thus, we can suppose that CDB-used fluorescent proteins are suitable for the reporter gene system to monitor the real status of gene expression and regulation.

Besides, in Figure 5, the mRNA variations between EGFP and EGFP-CDB or DsRed and CDB-DsRed are almost the same. Therefore, we can assume that CDB DNA fragment will not influence the transcription efficiency of its combined reporter genes. These results also demonstrate again that CDB affects the stability of fusion protein through a posttranslational regulation [8].

### 3.4. Relative Fluorescent Intensities and Induction Ratio of Curcumin-Induced Fluorescent Proteins

The CMV IE promoter is regulated by several transcription factors in human cells, which contains a number of 19 bp repeat elements and exhibits strong enhancer activity after binding the transcription factor, cAMP response element binding protein (CREB) [25, 26]. In addition, curcumin is having a modulatory effect on the transcription factor CREB expression [27]. Hence, for examination of the effect of CDB-fused fluorescent protein on induction condition, curcumin has been utilized as an inducer for CMV IE promoter to monitor the slight gene regulations in transfected cells. The cytotoxicity of curcumin on MeWo cells was confirmed by using a standard tetrazolium-based MTT assay (data not shown) to find the proper treatment concentration of curcumin. Our results demonstrated that within 10 *μ*M of curcumin there was no observed cytotoxicity on MeWo cells. Therefore, we used 5 *μ*M of curcumin in the promoter induction assay.

**Figure 6:**
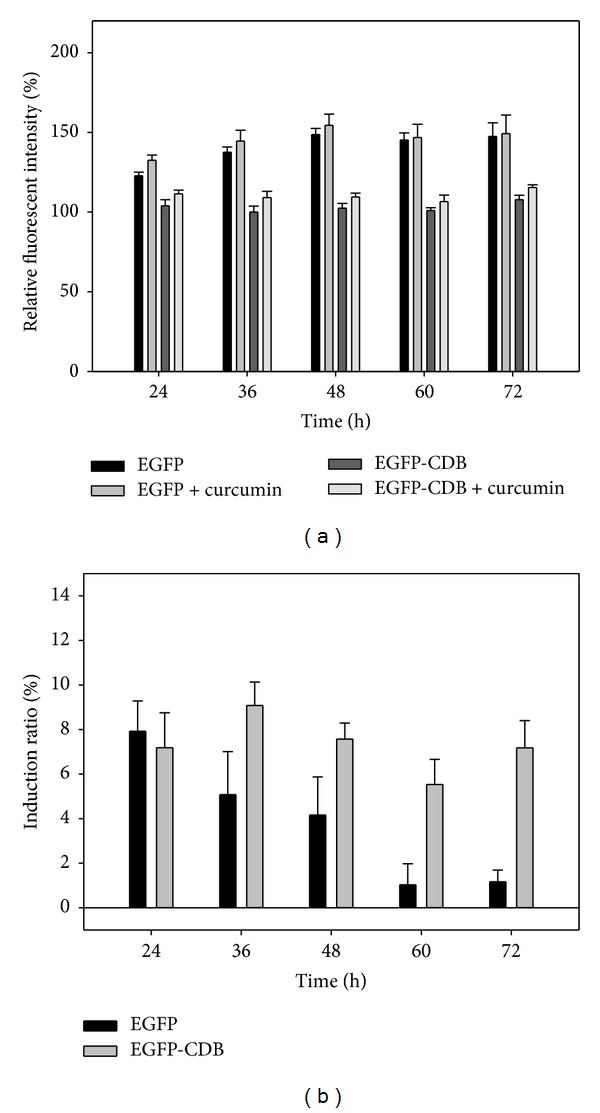
(a) Relative fluorescent intensities and (b) induction ratio of curcumin-induced pEGFP/pEGFP-CDB plasmids transfected MeWo cells at different time points.

**Figure 7:**
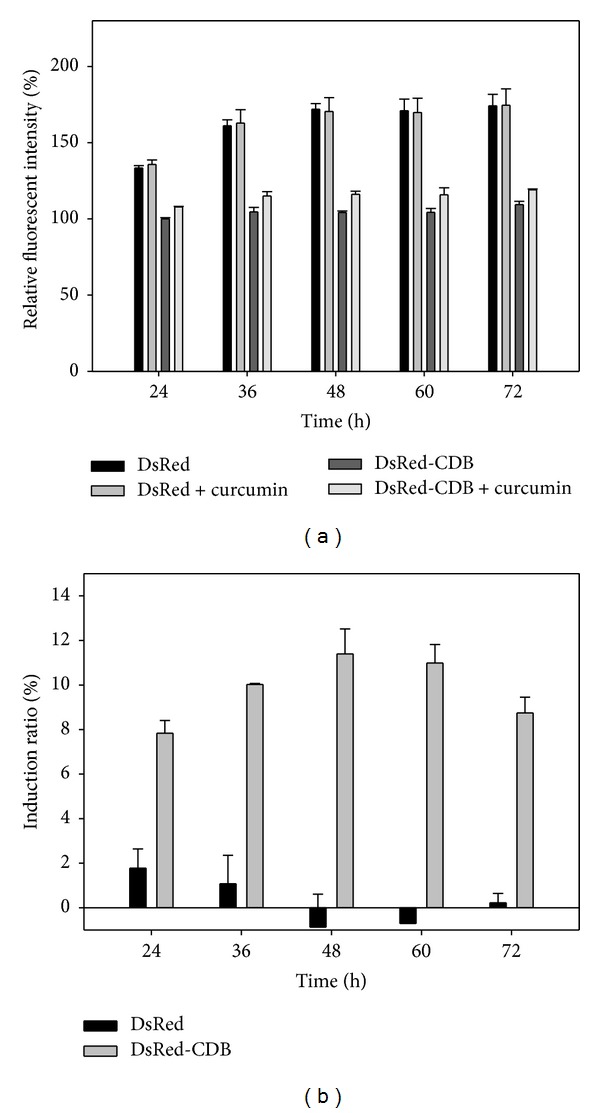
(a) Relative fluorescent intensities and (b) induction ratio of curcumin-induced pDsRed/pDsRed-CDB plasmids transfected MeWo cells at different time points.

These results were shown in Figures 6 and 7. For EGFP group, relative fluorescent intensities of pEGFP transfected cells with or without curcumin induction are only slightly dissimilar at 24 to 48. After 48 h transfection, the fluorescent intensities of EGFP group were nearly the same (Figure 6(a)). However, for EGFP-CDB group, the addition of curcumin can show the increase of fluorescent intensities at every analyzed time point in cells (Figure 6(a)). Moreover, in Figure 6(b), the calculated induction ratios for EGFP and EGFP-CDB was also demonstrated that CDB-fused fluorescent protein is able to monitor the minor regulations in the reporter gene system. The induction ratios of EGFP-CDB are approximately 6 to 10%. Besides, for DsRed and CDB-DsRed groups in Figure 7, the induction effect of curcumin on the CDB-DsRed group is evidently greater than that of DsRed group. The induction ratios of CDB-DsRed are approximately 8 to 12% at the induction time period. However, the expression of DsRed in curcumin-treated cells has only 1 to 2% induction ratio at 24 to 36 h and which is attenuated after 36 h (Figure 7(b)). This result might be caused by the high expression level of DsRed, which makes a great fluorescent intensity in transfected cells and then cause the induction effect become insignificant.

High stabilities of fluorescent reporter proteins interfere with the detection of rapid transient changes in gene expression. To avoid the disadvantages associated with fluorescent protein stability, destabilized constructs have been developed for use with mammalian cells and bacteria, including the use of PEST sequence and mouse ornithine decarboxylase (MODC) [20, 21, 28]. Therefore, we can suggest that this is a good strategy for improvement of fluorescent-protein-based reporter gene system and such modified fluorescent-protein-based reporter gene system can be utilized in the high throughput screening (HTS) of large chemical libraries to identify functional compounds [18].

In this study, we firstly confirmed the complete effect of human CDB on fluorescent-protein-based reporter gene system in human cells using simple constructions. Our results indicated that CDB-mediated protein instability can help the overly stable fluorescent proteins to be the proper reporters for the analyses of fast and transient gene regulations.

## 4. Conclusion

In conclusion, the original fluorescent proteins, both EGFP and DsRed, are stable and accumulated in the transfected cells. Alternatively, CDB-fused fluorescent proteins have instability because they will be destroyed with cyclins via ubiquitin-proteasome system during cell cycle. In addition, the CDB fragment can affect its fusion protein whether it is fused at N- or C-terminus. RT-PCR results demonstrated that the fluorescent intensities of CDB fusion proteins were almost reflected in their gene regulations. RT-PCR results also proved that CDB DNA fragment will not influence the transcription efficiency of its combined reporter genes. The curcumin-mediated gene induction experiments supposed that CDB-fused fluorescent protein will be able to examine the slight regulations in the reporter gene system. Therefore, we proposed that such modified fluorescent-protein-based reporter gene system has the potential to be utilized in the HTS system for screening of functional compounds in the future.

## Figures and Tables

**Table 1:** Primer sequences used for PCR and RT-PCR in this study.

Target	Type	Sequences
PCR		
Human CDB	Sense	5′-GCCGCGGACCATGGCCAACTCGAAAATTAATGCTGAAAAT-3′
	Antisense	5′-GGGATCCCGCTTCTTCATAGGCATTTTGGC-3′

RT-PCR		
EGFP	Sense	5′-GCATCGACTTCAAGGAGGAC-3′
	Antisense	5′-GAACTCCAGCAGGACCATGT-3′
DsRed	Sense	5′-GGACGGCTCCTTCATCTACA-3′
	Antisense	5′-GCTCCACGATGGTGTAGTCC-3′
GAPDH	Sense	5′-CCCTTCATTGACCTCAACTA-3′
	Antisense	5′-AGATGATGACCCTTTTGGCT-3′
